# Biphasic response of human iPSC-derived neural network activity following exposure to a sarin-surrogate nerve agent

**DOI:** 10.3389/fncel.2024.1378579

**Published:** 2024-09-05

**Authors:** Chandrakumar Bogguri, Vivek Kurien George, Beheshta Amiri, Alexander Ladd, Nicholas R. Hum, Aimy Sebastian, Heather A. Enright, Carlos A. Valdez, T. Nathan Mundhenk, Jose Cadena, Doris Lam

**Affiliations:** ^1^Physical and Life Sciences Directorate, Lawrence Livermore National Laboratory, Livermore, CA, United States; ^2^Engineering Directorate, Lawrence Livermore National Laboratory, Livermore, CA, United States; ^3^Global Security Directorate, Lawrence Livermore National Laboratory, Livermore, CA, United States

**Keywords:** multi-electrode array, human neuronal activity, organophosphate, nerve agent, acute exposure, human induced pluripotent stem cells, microphysiological systems

## Abstract

Organophosphorus nerve agents (OPNA) are hazardous environmental exposures to the civilian population and have been historically weaponized as chemical warfare agents (CWA). OPNA exposure can lead to several neurological, sensory, and motor symptoms that can manifest into chronic neurological illnesses later in life. There is still a large need for technological advancement to better understand changes in brain function following OPNA exposure. The human-relevant *in vitro* multi-electrode array (MEA) system, which combines the MEA technology with human stem cell technology, has the potential to monitor the acute, sub-chronic, and chronic consequences of OPNA exposure on brain activity. However, the application of this system to assess OPNA hazards and risks to human brain function remains to be investigated. In a concentration-response study, we have employed a human-relevant MEA system to monitor and detect changes in the electrical activity of engineered neural networks to increasing concentrations of the sarin surrogate 4-nitrophenyl isopropyl methylphosphonate (NIMP). We report a biphasic response in the spiking (but not bursting) activity of neurons exposed to low (i.e., 0.4 and 4 μM) versus high concentrations (i.e., 40 and 100 μM) of NIMP, which was monitored during the exposure period and up to 6 days post-exposure. Regardless of the NIMP concentration, at a network level, communication or coordination of neuronal activity decreased as early as 60 min and persisted at 24 h of NIMP exposure. Once NIMP was removed, coordinated activity was no different than control (0 μM of NIMP). Interestingly, only in the high concentration of NIMP did coordination of activity at a network level begin to decrease again at 2 days post-exposure and persisted on day 6 post-exposure. Notably, cell viability was not affected during or after NIMP exposure. Also, while the catalytic activity of AChE decreased during NIMP exposure, its activity recovered once NIMP was removed. Gene expression analysis suggests that human iPSC-derived neurons and primary human astrocytes resulted in altered genes related to the cell’s interaction with the extracellular environment, its intracellular calcium signaling pathways, and inflammation, which could have contributed to how neurons communicated at a network level.

## 1 Introduction

Organophosphorus nerve agents (OPNAs) remain one of the most toxic chemicals known to humankind, either chronically (e.g., pesticides) or acutely (e.g., sarin, soman, VX). The use of the latter in military conflicts such as the Iran-Iraq war served to highlight their mass destruction capabilities (Haines and Fox, 2014). Initially designed for use during military conflicts, it is their use against civilian targets (e.g., Syrian conflict, Tokyo subway attack) that has raised immediate concern among governments, which have taken more significant steps to ban their production and use (Abou-Donia et al., 2016; Dolgin, 2013). Abundant clinical evidence demonstrates that a single OPNA exposure can permanently alter normal human brain activity (Duffy et al., 1979; Sugiyama et al., 2020; Yanagisawa et al., 2006) and cause lasting somatic (e.g., fatigue, headaches, numbness in limbs) and psychological symptoms (e.g., insomnia, irritability, and forgetfulness) (Sugiyama et al., 2020). Abnormal electroencephalographic (EEG) or electrocorticographic (ECoG) recordings of brain activity have been observed in experimental rodent and non-human primate models at low or sub-lethal doses of OPNA (e.g., Soman, Sarin, NIMP) that persist 12–15 months after exposure (Angrand et al., 2021; Bloch-Shilderman et al., 2008; Crouzier et al., 2004; Pearce et al., 1999). These results are consistent with reports from victims of the Tokyo subway attack in 1994 and 1995, who also showed abnormal EEGs (Yanagisawa et al., 2006). Despite the extensive and supportive evidence from the neural monitoring technologies (e.g., EEG and ECoG), long-term (e.g., days to months) and reliable monitoring of brain activity (from the same neuron/networks) remains a major challenge. This leaves many unanswered questions in the OPNA field, in particular, how have principal neuronal populations been altered during and following OPNA exposure to generate this abnormal brain activity and what are the mechanisms of OPNA that can lead to permanent changes in brain function from a single acute exposure.

The functional consequences of OPNA on neural activity have mainly been studied using brain tissue slices of experimental animal models (e.g., rat or guinea pig) that have been exposed to the nerve agent for a period of time or were bath perfused (Anderson et al., 2021; Endres et al., 1989; Harrison et al., 2005; Thinschmidt et al., 2022; Kozhemyakin et al., 2010). Extracellular recordings or whole-cell patch clamping techniques have revealed increased excitability of individual neurons isolated from the hippocampus or basolateral amygdala as described by the increased frequency, and, in some studies the amplitude, of spontaneous excitatory postsynaptic currents (sEPSC) when exposed to soman, sarin, or paraoxon (Thinschmidt et al., 2022; Kozhemyakin et al., 2010; Alkondon et al., 2013). Moreover, a concentration-dependent response has been described for paraoxon and the sarin-surrogate, 4-nitrophenyl isopropyl methylphosphonate (NIMP) (Kozhemyakin et al., 2010; Thinschmidt et al., 2022). However, it also remains unclear how changes in neuronal activity affect communication at a network level.

An *in vitro* multi-electrode array (MEA) system, which integrates the MEA technology with human stem cell technology, can mimic key structural and functional features of the human brain *in vitro* (Haring et al., 2017; Koo et al., 2018; Pamies et al., 2017). This system provides an unprecedented means of non-invasively monitoring fast neuronal dynamics from human-induced pluripotent stem cell (iPSC)-derived neurons at a single cell, network (i.e., the communication between two neurons), and community level (i.e., communication within a cluster of networks) with increasing spatiotemporal precision for a prolonged period of time (Anderson et al., 2021; Bang et al., 2019). In this study, we conducted a concentration-response experiment to evaluate the functional consequence of human-relevant neural and network activity (comprised of human-iPSC derived glutamatergic and GABAergic neurons) to an increasing concentration of the sarin surrogate NIMP. NIMP is a non-volatile compound that is stable in aqueous solution and known to inactivate the enzyme acetylcholinesterase (AChE) in a similar manner as sarin (Meek et al., 2012; Ohta et al., 2006; Bennion et al., 2021). Our findings demonstrate, for the first time, the application of a human-relevant MEA system in real-time monitoring of brain-like activity from engineered human-iPSC-derived neural networks at the onset and duration of NIMP exposure and days to a week after exposure. Furthermore, we describe a concentration-dependent biphasic response in spiking, but not bursting, activity that becomes less coordinated at a network level: (1) hyperexcitability in spiking activity during exposure to low (0.4 and 4 μM) but not high (40 and 100 μM) concentrations of NIMP; and (2) decline in spiking activity post-exposure only in the high concentrations of NIMP. Interestingly, networks continue to exhibit less coordinated activity after the removal of NIMP only in the high concentration condition, even after spiking activity returned to normal levels by 6 days post-exposure. The changes in spiking and network activity during days following exposure to a high concentration of NIMP were not attributed to restored levels of AChE activity (determined using Ellman’s assay) or cell viability (determined using the lactate dehydrogenase assay), but instead suggest more fundamental cellular changes at the transcriptomic level (as evidenced by our transcriptomic profiling) affecting the function of neurons and astrocytes within the MEA system.

## 2 Materials and methods

### 2.1 Chemicals

The sarin surrogate nitrophenyl isopropyl methylphosphonate (NIMP) was synthesized according to previously documented methods (Meek et al., 2012). For concentration-response experiments, 50% of the culture media was removed and replaced with a 2X concentrated NIMP solution for a final concentration of 0.4, 4, 40, and 100 μM, concentrations selected from a pilot study and within range of previous work (Thinschmidt et al., 2022).

### 2.2 Cell culture

Human iPSC-derived glutamatergic and GABAergic neurons (at a ratio of 70:30) and primary human astrocytes (Neucyte, San Jose, CA, USA) were co-cultured, at a ratio of 75:25 neurons to astrocytes (total 2,835 cells/mm^2^). The ratio was informed by the vendor’s recommendation in previous studies conducted by our group (Lam et al., 2023) and others (Sasaki et al., 2019; Wang et al., 2023). As described previously (Lam et al., 2022), 6-well layout MEA devices (MEA200/30iR-ITO, Multi-channel systems, Reutlingen, Germany) were plasma-treated (PDC-001-HP, Harrick Plasma) then soaked in PBS overnight at 30°C. The MEA devices were washed with sterile DI water (4X) and then air-dried before autoclaving at 121°C. Both MEA devices and 96 flat bottom-well plates were coated with 0.1% PEI (prepared in borate buffer) for overnight incubation at 37°C, washed with sterile DI water (4X), then coated with 20 μg/ml of laminin for 2 h at 37°C. Laminin was removed before cells were seeded. Specific ratios for neuronal subtypes (e.g., glutamatergic and GABAergic neurons) and cell types (e.g., neurons to astrocytes) were obtained from purified stocks. Cells were resuspended in seeding media (Neucyte) based on live cell concentrations then introduced into the MEA device or 96-flat bottom-well plate. Cultures were maintained in a humidified incubator (37°C, 5% CO_2_). After 24 h, media was replaced with short term media (Neucyte) for 1 week before switched to long term media (Neucyte) for the duration of the experiment. For culture maintenance, 50% of media was replaced every 2–3 days.

### 2.3 Multi-electrode array (MEA) recording

A 256-channel MEA2100 recording system (Multichannel Systems, Reutlingen, Germany) was used to record electrophysiology activity for 30 min at a sampling frequency of 10 kHz and bandpass filtered between 4 and 4,000 Hz, as before by our group (Enright et al., 2020; Lam et al., 2019; Soscia et al., 2017; Soscia et al., 2020) and others (Novellino et al., 2011; Pastore et al., 2018). This involved the placement of the 6-well MEA device within a 5% CO_2_-regulated chamber on the heated stage (37°C), and electrophysiology recording started after a standard 5-min equilibration time. An action potential spike was defined by a lower limit threshold, set at 6.5x the standard deviation of baseline noise, for each electrode. Devices were recorded for 30 min once a week from 7 days *in vitro* (DIV) and onward to monitor the development and maturation of neural networks. Qualitative counts of the percentage of active electrodes for each device were monitored by the experimenter on the day of recording. By ∼25 DIV, a time point in which the number of active electrodes has stabilized (Supplementary Figure 1), baseline activity of the 6-well MEA device was recorded and then wells were randomly assigned for NIMP treatment (0, 0.4, 4, 40 and/or 100 μM). To our knowledge, NIMP exposure studies in *in vitro* models are scarce. The concentrations of NIMP used in the present study were determined from a pilot *in vitro* study that had used 0.4 and 4 μM of NIMP. We extended the concentration range to include 10X and 25X the highest concentration and included 40 and 100 μM. These concentrations were also in range with recently published work on acute NIMP exposure in brain slices (Thinschmidt et al., 2022) and cell culture exposure to insecticides (van Melis et al., 2023; van Melis et al., 2024). For the 0 μM NIMP condition, culture media was added to the wells to mimic the mechanical perturbation of dosing the cultures. Following treatment with NIMP, recordings were conducted within the first hour of exposure (i.e., 2 × 30 min recordings), and 1-, 2-, and 6 days post-exposure.

#### 2.3.1 MEA data analysis

As in previous studies (Lam et al., 2022; Enright et al., 2020; Lam et al., 2019; Soscia et al., 2020), time-stamped data from each recording is exported as an HDF5 file and analyzed using an in-house custom R package; burst detection parameters similar to those of Charlesworth et al. (2015) and Chiappalone et al. (2006) were defined. Specifically, the following values were used: maximum beginning interspike interval (ISI) of 0.1 s, maximum end ISI of 0.2 s, minimum interburst interval (IBI) of 0.5 s, minimum burst duration of 0.05 s, and minimum number of spikes per burst of 6. Additional parameters in this study included the removal of any electrodes with a mean burst duration greater than 5 s to eliminate potentially noisy electrodes. To capture electrodes that fully ceased activity following NIMP exposure, electrodes were considered active in the time-course study if spiking activity was detected across 3-time points: at baseline, 30 min, and 60 min of NIMP exposure. For electrodes within an array of a well that had no detectable spiking or bursting activity at later time points, a value of “0” was included for that electrode in the final analysis. To minimize the effect of mechanical disturbance, attributed to pipetting in the NIMP solution, the mean (for a specific feature) before NIMP exposure (e.g., baseline) was calculated. Then, the mean following NIMP exposure was calculated and normalized to baseline activity. The values for the NIMP-treated wells (normalized to baseline) are expressed as a fold change difference to the average value from the 0 μM NIMP cultures (normalized to baseline) and used for further statistical analysis.

#### 2.3.2 Synchrony and network analysis

Synchrony analysis was performed using the SPIKE distance (Kreuz et al., 2013), as previously described (Lam et al., 2023; Lam et al., 2022; Enright et al., 2020; Lam et al., 2019). The SPIKE-distance between two spike trains is the average of the *instantaneous* dissimilarity between the two spike trains at different points of the recording. Values were subtracted from 1 to obtain a similarity or synchrony measure, such that a value of 1 represents completely synchronous firing and a value of 0 denotes asynchrony. Additionally, values were normalized by the SPIKE-distance obtained on randomly generated spike trains to compensate for the documented bias of SPIKE distance to assign lower synchrony values as the ratio of the number of spikes between two spike trains moves away from 1. To remove potentially noisy electrodes, electrodes with a mean burst duration greater than 5 s were excluded from the analysis. Less than 1% of electrodes across all experiments were removed by this criterion.

Quantile-Quantile (QQ) plots (Wilks, 2019) and the Kullbach–Leibler (KL) divergence (Kullback and Leibler, 1951) are used to visualize and quantitatively describe the synchrony changes across experimental conditions. A QQ plot is a non-parametric method to compare two probability distributions. The qqplot_2samples function from Python’s statsmodels library version 0.14.1 was used for the calculations. If the two distributions are the same, then points will lie on a straight line with a unity slope. To calculate the KL divergence, the kl_div function in Python’s Scipy library was used. When two distributions are the same the KL divergence is zero; Values greater than zero indicate deviations between the distributions—i.e., differences between the histograms of the empirical data. To estimate the KL divergence, synchrony values were discretized into 100 evenly-spaced bins.

### 2.4 Ellman’s assay

AChE can exist as both a soluble and membrane-bound form. Previous studies have detected enzymatic activity from the soluble form in the culture supernatant obtained from HEK293 cells (Velan et al., 1991) and neuronal cultures (Dong et al., 2004; Xiang et al., 2014). We have also confirmed the presence of active human AChE in the culture media from the human-relevant MEA system, according to a modified Ellman’s method (Ellman et al., 1961), which included the addition of 0.5 mM tetraisopropyl pyrophosphoramide (iso-OMPA), an inhibitor of butyrylcholinesterase, for 15 min at 37°C. The culture supernatants were diluted 1:20 into the substrates acetylthiocholine (1 mM) and DTNB (1 mM). Enzyme activity was examined by measuring the optical density (OD) at an absorbance of 410 nm at 25°C using Synergy H1 multi-mode microplate reader (BioTek). OD was recorded in 5-min intervals over the course of 2 h. The rate of change in enzymatic activity (based on OD) over 2 h for cultures treated with NIMP was normalized to the rate of change in enzymatic activity in the 0 μM NIMP culture. Total cellular protein for Ellman’s assay was conducted based on a previous method neurons and non-neuronal cells (Thullbery et al., 2005), cultures were washed with PBS (3x) and cells lysed using 1% Triton X-100 in 50 mm Tris (pH 8.0) and 150 mM NaCl. Then the cell lysate was treated with DTNB (final concentration 0.32 mM) and iso-OMPA (final concentration, 0.01 mM) for 10–15 min at 37°C. After the incubation period, ATCH (final concentration, 0.75 mM) was added before samples were read on the plate reader as described for the culture supernatant.

### 2.5 Cell viability assay

CyQuant™ Lactate Dehydrogenase (LDH) assay (Thermo Fisher Scientific) was performed on culture supernatants collected at the 24-h time point of NIMP exposure and 6- days post-exposure. Briefly, the supernatant was collected from 0 μM or NIMP-treated (0.4, 4, 40, and/or 100 μM) cultures and processed in 96-well plate format, per kit instructions. Absorbances were read at 490 and 680 nm on the Synergy H1 multi-mode microplate reader (BioTek), and absorbance data for samples exposed or having been exposed to NIMP were normalized to the 0 μM NIMP condition.

### 2.6 Bulk sequencing

Cultures were treated with NIMP (0, 4, and 100 μM) for 24 h, and cells were lysed at 6 days post-exposure using RLT buffer containing β-mercaptoethanol; the supernatant was collected at 6 days post-exposure as well. Total RNA was extracted and purified from the collected supernatant using the RNAeasy mini spin columns (Qiagen). Sequencing libraries were prepared using Illumina Stranded mRNA Prep (Illumina, San Diego, CA, United States) and sequenced using an Illumina NextSeq 2000. The quality of sequencing data was checked using FastQC software.^1^ The reads were then mapped to the human genome (hg38) using STAR aligner and read counts per gene were determined using “featureCounts” from Rsubread package (Liao et al., 2014). RUVseq was then used to identify and remove factors of unwanted variation (Risso et al., 2014). Differentially expressed genes were then identified using edgeR, controlling for factors of unwanted variation (Robinson et al., 2010). A gene was considered “significantly differentially expressed” when its false discovery rate adjusted *p*-value was < 0.05 and fold change was > 1.5. Gene ontology (GO) analysis was performed using ToppGene (Chen et al., 2009). Heatmaps were generated using heatmap.2 function in “gplots” R package. Volcano plots were generated using Galaxy Europe.^2^

### 2.7 Statistical analysis

Quantified data are expressed as mean ± standard error of the mean (SEM) for the number of wells indicated unless stated differently. For electrophysiology experiments and *in vitro* assays, the statistical significance was analyzed in GraphPad version 10 (GraphPad Software, San Diego, CA) using unpaired *t*-test, mixed model repeated measures for one-way or two-way ANOVA with Tukey’s or Dunnett’s post-hoc analysis.

## 3 Results

### 3.1 Functional characterization of human iPSC-derived neural and network activity during and following NIMP exposure

**FIGURE 1:**
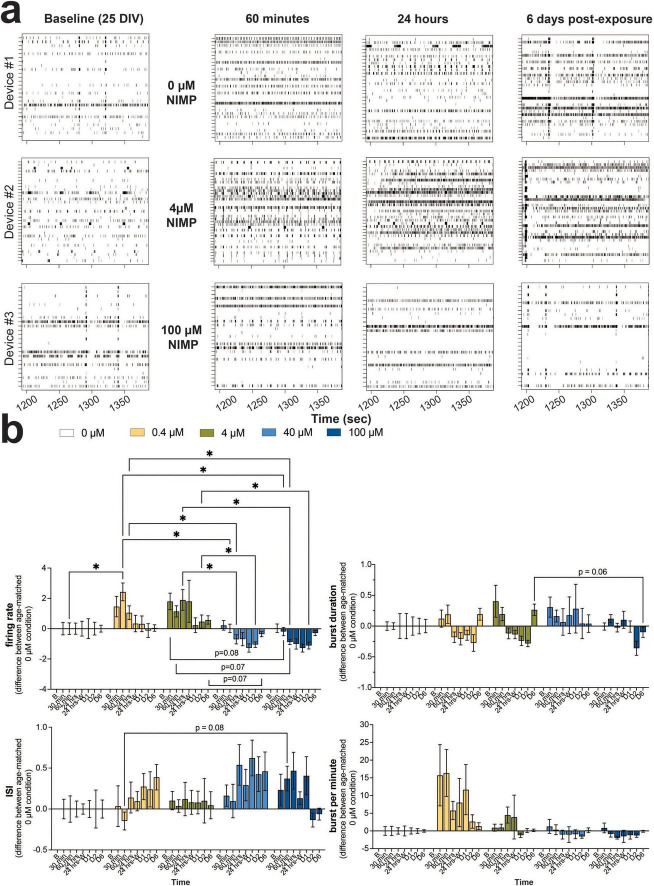
Concentration-dependent changes in neural and network activity from human iPSC-derived neurons co-cultured with primary human astrocytes in the human-relevant MEA system during 24 h of NIMP exposure and up to 6 days post-exposure. **(a)** Representative 3-min raster plot showing spiking and bursting activity for each electrode (row) before (i.e., baseline), during (i.e., 60 min, 24 h), and after (i.e., 6 days) NIMP exposure. **(b)** Bar graph summarizes the features of spiking (i.e., firing rate, interspike interval) and bursting (i.e., burst duration, burst per minute) before (i.e., baseline [b]), during (i.e., 30 and 60 min, 24 h), and after (i.e., immediately after washout [24 h-W], 1-[D1], 2-[D2], and 6-[D6] days) NIMP (i.e., 0, 0.4, 4, 40, and 100 μM) exposure. Data is shown as the difference in mean value for the treatment condition relative to the age-matched 0 μM condition, shown as mean ± SEM for *n* = 7–13 wells/ treatment condition. Statistical analysis was conducted using mixed model repeated measure two-way ANOVA with Tukey’s *post-hoc* test to compare age-matched treated and 0 μM conditions (*). Statistical significance is shown at a level of *p < 0.05.

The consequence of OPNA can have widespread effects on the brain. Thus, in the present study, we modeled the human-relevant MEA system after the cortex, containing glutamatergic (excitatory) neurons (70–80%) and GABAergic (inhibitory) neurons (20–30%) (Markram et al., 2004; DeFelipe and Farinas, 1992). Based on our previous work in a 3D MEA study (Lam et al., 2023), in other 2D MEA studies (Sasaki et al., 2019; Wang et al., 2023), and recommended by the vendor, the seeding ratio of 70:30 human iPSC-derived glutamatergic to -derived GABAergic neurons was co-cultured with primary human astrocytes (at a ratio of 25:75 astrocytes to neurons) on the planar 6-well MEA device. As the number of active electrodes plateaued by 25 DIV (Supplementary Figure 1), spiking and bursting activity appeared coordinated (or synchronized) across a pair and/or multiple electrodes, as shown by the representative raster plot (Figure 1a) that displays spiking and bursting activity before NIMP exposure (i.e., baseline). The human-relevant MEA system (∼25 DIV) was selected at random and treated with one of five concentrations of NIMP (0, 0.4, 4, 40, and 100 μM) for 24 h. Representative raster plots (Figure 1a) display spiking and bursting activity during the period of exposure to NIMP, specifically at 60 min and 24 h during NIMP exposure, and 6 days after NIMP has been removed relative to age-matched 0 μM NIMP cultures. Qualitative observations from the raster plots suggest that cultures exposed to a low concentration of NIMP (4 μM) exhibited more uncoordinated spiking activity across electrodes during NIMP exposure (e.g., 60 min and 24 h) that appeared to be resolved once NIMP was removed (e.g., 6 days post-exposure). Cultures exposed to a high concentration of NIMP (100 μM) displayed greater coordination of spiking and bursting activity across multiple electrodes at 60 min of exposure that became less coordinated by 24 h of exposure. By 6 days post-exposure, less activity was observed in cultures treated with a high concentration of NIMP compared to age-matched 0 μM NIMP cultures.

We quantitatively assessed the features of spiking (Figure 1b, left column) and bursting activity (Figure 1b, right column) across NIMP concentrations, and time points were quantified and normalized to baseline activity for each treatment-matched culture to minimize the effect of mechanical perturbation by the addition of NIMP. Then, the fold change difference between age-matched treatment wells was calculated. A value of “0” indicates no difference in activity of the NIMP-treated culture relative to the 0 μM NIMP culture. Time series analysis of neural activity revealed that NIMP altered only two features of spiking activity and had no effect on the features of bursting activity for human-iPSC neurons within the cell culture system (Figure 1 and Supplementary Figure 2). For cultures treated with a low concentration of NIMP (0.4 or 4 μM), significant increases in the firing rate (Figure 1b) and total number of spikes (Supplementary Figure 2) were observed for the duration of NIMP exposure, as early as 60 min for 0.4 μM (*p* < 0.05) and 30 min for 4 μM NIMP (*p* < 0.05). Increased excitability persisted at 24 h of NIMP exposure for 4 μM NIMP (*p* < 0.05); this excitability returned to levels comparable to the 0 μM NIMP cultures when NIMP was immediately removed and was sustained at 6 days post-exposure for both low concentrations of NIMP. Cultures treated with a high concentration of NIMP (40 or 100 μM), however, showed a significant decline in the firing rate and total number of spikes at 24 h of NIMP exposure (*p* < 0.05) that persisted at 2 days post-exposure (*p* < 0.05) when compared to age-matched cultures treated with a low concentration of NIMP. This decline in activity was not significantly different when compared to age-matched 0 μM NIMP cultures. Differences between a low versus high concentration of NIMP were observed or trending toward statistical significance for specific time points. For example, a significant decrease in the percentage of active electrodes was detected at 6 days post-exposure (*p* < 0.05, Supplementary Figure 2) or interspike interval at 60 min (*p* = 0.08) and burst duration at 6 days post-exposure (*p* = 0.06) showed opposite regulation of a feature of spiking and bursting activity (Figure 1b). No detectable differences were observed for features of bursting activity (e.g., burst per minute in Figure 1 or the total number of bursts and interburst interval in Supplementary Figure 2). Notably, the concentration-dependent effect observed for the firing rate and the total number of spikes were similar in neuronal excitability for cultures treated with 0.4 and 4 μM NIMP and similarly the level of activity was reduced for cultures treated with 40 and 100 μM. In this paper, here on out, we have categorized cultures exposed to 0.4 and 4 μM as “low” versus 40 and 100 μM NIMP as “high” concentrations of NIMP.

**FIGURE 2:**
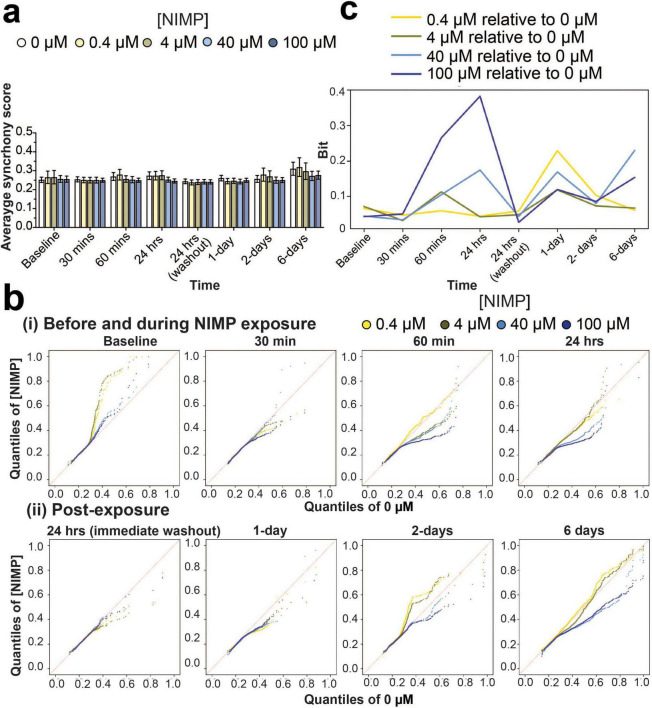
Synchrony analysis of network activity before, during, and after NIMP exposure. **(a)** Bar graph summarizes the average synchrony score (mean ± SEM) from a 30 min recording for networks within the human-relevant MEA systems exposed to 0 μM (*n* = 12 devices), 0.4 μM (*n* = 7 devices), 4 μM (*n* = 8 devices), 40 μM (*n* = 11 devices), and 100 μM (*n* = 13 devices). **(b)** Quantile-Quantile (QQ) plots display the synchrony scores for networks aggregated across all human-relevant MEA system for each time point before and during NIMP exposure (i), and post-exposure (ii). Data is shown for 0.4 (yellow dot), 4 (green dot), 40 (light-blue dot), and 100 (dark-blue dot) μM NIMP with 0 μM as the reference line in red. **(c)** Line graph displays the Kullback-Leibler (KL) divergence that quantifies the statistical distance (in Bits) from Supplementary Figure 3 between the probability distribution of synchrony scores obtained from aggregated data of human-relevant MEA system at each time point exposed to 0.4 (yellow line), 4 (green line), 40 (light blue line), or 100 (blue line) μM NIMP relative to the 0 μM NIMP condition, the reference distribution.

We examined the degree of coordination (or synchrony) in spike timing between electrodes for networks within the human-relevant MEA system. As before (Lam et al., 2022; Enright et al., 2020; Lam et al., 2019), the SPIKE distance method (Kreuz et al., 2013) was used to score all possible electrode pairings for a given 30-min recording for each MEA system (Lam et al., 2022; Enright et al., 2020; Lam et al., 2019). A score of “0” indicates that the network has no synchrony and a score closer to “1” indicates a high degree of synchrony. At a device level, synchrony analysis revealed that the average score for networks exposed to all concentrations of NIMP (e.g., 0.4, 4, 40, and 100 μM) were no different from the 0 μM NIMP condition at any of the time points during and after NIMP exposure (Figure 2a), which ranged from 0.24 to 0.31. However, when we evaluated the distribution of synchrony scores for all networks (aggregated across all devices) grouped by NIMP-treated condition and each time point, we observed differences in a subpopulation of networks that were masked when analysis was conducted at a gross level.

The distribution of synchrony scores is reported in a histogram (Supplementary Figure 3) and shown as a probability distribution segregated into quantiles in the quantile-quantile (QQ) plot (Figure 2b). At each time point, to generate the QQ plots we used the data from all wells for each concentration. At baseline, synchrony scores are shown to be variable across multiple devices before being selected for NIMP treatment, having networks with synchrony scores within quantiles that range from 0.1 to 1 (in both *x* and *y* axes, Figure 2bi). At 30 and 60 min of NIMP exposure, synchrony scores from human iPSC-derived networks at 4, 40, and 100 μM concentration showed that a subset of networks decreased its synchrony score, as shown by a decrease in the quantiles that range from 0.1 to 0.7 in the *y*-axis, having scores below the 0 μM NIMP condition (red line). By 24 h, a greater subset of networks exhibited a decrease in synchrony scores in the 40 and 100 μM NIMP concentrations, while the 0.4 and 4 μM concentrations displayed a distribution of synchrony scores comparable to 0 μM concentration.

Interestingly, despite the removal of NIMP and the replacement of fresh media, the distribution of synchrony scores for networks previously treated with NIMP, independent of its concentration, had decreased at 1-day post-exposure relative to age-matched networks treated with 0 μM NIMP. By 2 days post-exposure, concentration differences were observed in the human-relevant MEA system previously treated with 0.4, 4, 40, and 100 μM NIMP. Increased coordinated activity was detected in a subset of networks (within the 0.4–0.8 quantiles) from the human-relevant MEA system exposed to lower NIMP concentrations (at 0.4 and 4 μM) that returned to levels exhibited by the age-matched cultures treated with 0 μM NIMP by 6 days post-exposure. Conversely, for the human-relevant MEA system exposed to high concentrations of NIMP (at 40 and 100 μM), a decrease in coordinated activity was observed for a subset of networks (within the 0.4–0.8 quantiles) observed at 2 and 6 days post-exposure.

For quantitative analysis, we used Kullback–Leibler (KL) divergence to determine the statistical distance from the probability distribution of synchrony scores (from Supplementary Figure 3) obtained from the MEA system exposed to 0, 0.4, 4, 40, and 100 μM NIMP compared to the 0 μM NIMP condition (the reference distribution) (Figure 2c), thereby quantifying the concentration-dependent effect of NIMP at each time point. In using this analysis, we observe that the coordinated activity for a subset of networks is affected for the duration of NIMP exposure for all concentrations of NIMP. At the 24-h time point, the magnitude of NIMP’s effect on network activity is greatest for 100 μM, and there is a gradual concentration-dependent decrease in response. However, at 6 days post-exposure, the 40 and 100 μM NIMP conditions’ distributions start to deviate from 0 μM condition’s distribution, relative to 0.4 and 4 μM NIMP conditions’ distributions. This suggests that a single exposure to a high concentration of NIMP can have lasting effects on subsets of neurons and their networks, detected days after NIMP exposure.

### 3.2 Monitoring AChE catalytic activity and cell viability during and after NIMP exposure

**FIGURE 3:**
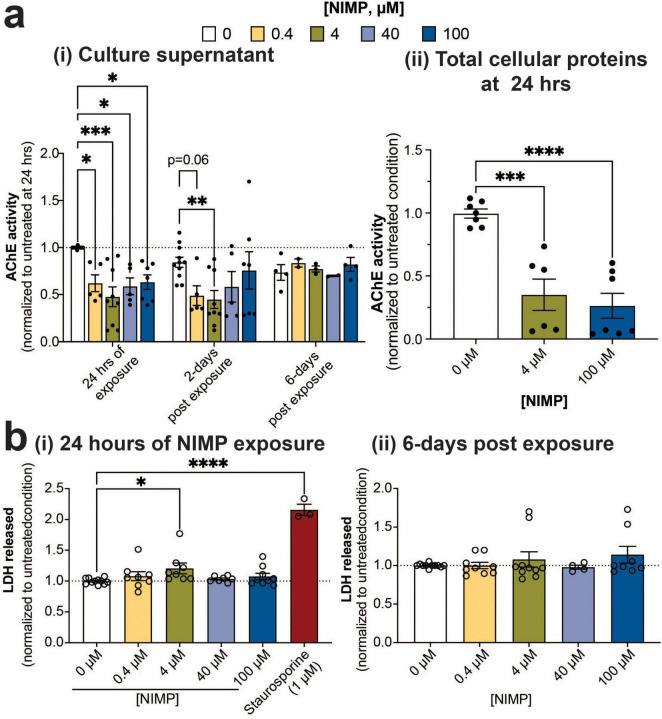
AChE expression and catalytic activity and cell viability. **(a)** Bar graph summarizes Ellman’s assay of acetylcholine hydrolysis activity by AChE from culture supernatants **(i)** obtained from 0 μM and NIMP-treated (i.e., 0.4, 4, 40, and 100 μM) cultures at 24 h of exposure and 2- and 6 days post-exposure. Data (mean ± SEM, *n* = 5–11 technical replicates/ concentration/time point conducted across 2–3 biological replicates) from the NIMP-treated group was normalized to the 0 μM group at 24 h of exposure (dotted line at 1). In addition, acetylcholine hydrolysis activity from the total cellular proteins collected at the 24-h time point of exposure from 0 μM and NIMP-treated (i.e., 4, and 100 μM) cultures is summarized **(ii)**. Data is shown as mean ± SEM, *n* = 2–3 technical replicates/ concentration across 2 biological replicates from the NIMP-treated group was normalized to the 0 μM group at 24 h of exposure. **(b)** Bar graph summarizes the amount of LDH released in the supernatant of 0 μM and NIMP-treated cultures normalized to the age-matched 0 μM NIMP condition (dotted line at 1). LDH was assessed during 24 h of NIMP exposure **(i)** and at 6 days post-exposure **(ii)**. Data is shown as mean ± SEM with *n* = 7–11 technical replicates/ treatment conditions from 4 biological replicates. Statistical analysis in this figure was conducted using mixed-model repeated measure (for **ai**) or one-way ANOVA (for **aii** and **b**) with Dunnett’s *post-hoc* test to compare between age-matched NIMP-treated and 0 μM NIMP condition. Statistical significances were observed at a level of *p < 0.05, **p < 0.01, ***p < 0.001, and ****p < 0.0001.

Exposure to OPNA is known to induce a “cholinergic” crisis in the central and peripheral nervous system (Franjesevic et al., 2019). OPNA-induced inhibition of AChE prevents the hydrolysis of acetylcholine and results in the accumulation of the neurotransmitter in the synapses and the overstimulation of nicotinic and muscarinic acetylcholine receptors (Franjesevic et al., 2019). To evaluate whether the electrophysiological changes during and after exposure to NIMP paralleled the inhibition of AChE activity, we examined the catalytic activity of the soluble form of AChE from the culture supernatant at the 24-h time point of NIMP exposure, and at 2- and 6- days post-exposure (Figure 3ai). All concentrations of NIMP showed reduced AChE catalytic activity (38–52% decrease) within the MEA system at the 24-h time point, but only the 4 μM-NIMP-treated MEA system appeared to show a sustained decrease in AChE activity at 2 days post-exposure, relative to age-matched 0 μM NIMP-treated human-relevant MEA system. By 6 days post-exposure, AChE activity for NIMP-treated cultures were no different to age-matched 0 μM NIMP-treated cultures. We also assessed the catalytic activity of the membrane-bound form of AChE from the total cellular proteins extracted from the MEA system at the 24-h time point for NIMP exposure (Figure 3aii). A greater reduction of AChE activity to 35% for 4 μM and 26% for 100 μM NIMP was observed. Thus, we detected the reduction of AChE activity during NIMP exposure and the recovery of its activity post-exposure using the human-relevant MEA system. However, the time-dependent electrophysiological changes observed in the low versus high concentrations of NIMP during and after exposure suggests that spiking and synchronized network activity are not solely dependent on NIMP-induce AChE inhibition. We also examined whether the observed changes in neural activity from the human-relevant MEA system were attributed to cell health. Cell viability was quantified by the amount of LDH released from the human-relevant MEA system exposed to NIMP at the 24 h time point (Figure 3bi) and 6 days post-exposure (Figure 3bii). LDH is a cytoplasmic enzyme that is released from cells with compromised membrane integrity, a feature of cells undergoing cell death (e.g., apoptosis, necrosis). As a positive control, cultures were treated with staurosporine (1 μM) to induce cell death (Moors et al., 2009). Despite having been treated with NIMP, all concentrations showed no change in cell viability, relative to the 0 μM NIMP condition, at either time point assessed. Collectively, these findings suggest that the functional changes observed in neural and network activity (e.g., features of spiking activity and synchrony) were either environmental or intrinsic changes to the cell.

### 3.3 Transcriptomic profiling of the human-relevant MEA system after NIMP exposure

**FIGURE 4:**
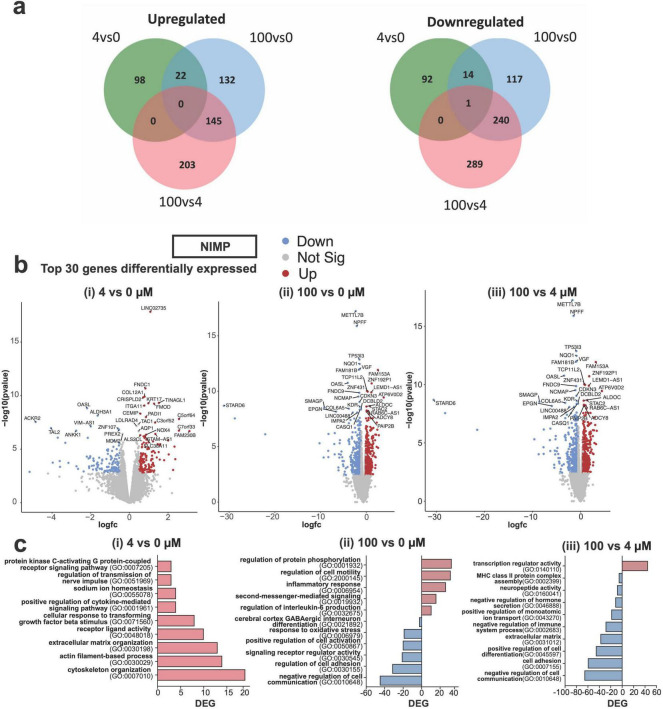
Differential gene expression from human iPSC-derived neurons co-cultured with primary human astrocytes at 6 days post NIMP exposure. **(a)** Venn diagram displays the number of shared or different genes upregulated (left) or downregulated (right) between treatment conditions: 4 vs. 0 μM, 100 vs. 0 μM, and 100 vs. 4 μM NIMP. **(b)** Volcano plots of statistical significance versus the magnitude of gene expression change between treatment conditions are shown for 4 vs. 0 μM **(i)**, 100 vs. 0 μM **(ii)**, and 100 vs. 4 μM **(iii)** NIMP. **(c)** Gene Ontology (GO) categories for genes upregulated and/or downregulated and are unique to 4 vs. 0 μM **(i)**, 100 vs. 0 μM **(ii)**, and 100 vs. 4 μM **(iii)** NIMP.

Having observed differences in the functional responses of neural and network activity from human-iPSC derived glutamatergic and GABAergic neurons exposed to a low versus high concentrations of NIMP, we examined whether transcriptomic changes within the human-relevant MEA system could provide insight on whether and how changes in the cellular environment or its molecular machinery affected neuronal function. Transcriptomic analysis was conducted on human-relevant MEA system at 6 days post-exposure to 0, 4, and 100 μM NIMP. Our analysis identified 227 differentially expressed genes (DEGs) in our human-relevant MEA system exposed to 4 μM NIMP relative to 0 μM NIMP versus 671 genes in our MEA system exposed to 100 μM NIMP relative to the 0 μM NIMP condition. When comparing 100 μM NIMP relative to 4 μM NIMP, 878 genes were found to be differentially expressed. As shown in the Venn diagram (Figure 4a), a greater proportion of genes were differentially expressed and unique to the 100 μM NIMP condition, as indicated by the lack of overlap between the 4 vs. 0 μM NIMP and 100 vs. 4 μM NIMP (green and red circles, respectively).

The top 30 genes differentially expressed are shown in the volcano plots [DEGs; abs (Fold Change, FC) > 1.5 and False discovery rate (FDR) < 0.05] for each comparison (Figure 4b). Ontology enrichment analysis of upregulated genes from 4 μM NIMP vs. 0 μM NIMP condition had identified biological processes associated with the extracellular environment and interaction with the cells (e.g., cytoskeleton organization, actin-filament-based process, extracellular matrix organization), nerve signals (e.g., sodium ion homeostasis, regulation of transmission of nerve impulses), and receptor ligand activity and signaling pathways (e.g., cytokines, PKC-activating G protein-coupled receptor) as enriched (Figure 4ci). Genes downregulated in 4 μM NIMP relative to 0 μM NIMP condition did not cluster into known biological processes. For 100 μM NIMP relative to 0 μM NIMP condition, upregulated genes were involved in biological processes that included the regulation of specific cellular functions (e.g., cell motility and inflammatory response), and intracellular signaling (e.g., protein phosphorylation, and second messenger-mediated signaling) (Figure 4cii). Biological processes associated with the downregulated genes in 100 μM NIMP relative to 0 μM NIMP condition included negative regulation cell communication, cell adhesion, positive regulation of cell activation, oxidative stress, cerebral cortex GABAergic interneuron differentiation and signaling receptor regulator activity. When comparing DEG in 100 μM NIMP relative to 4 μM NIMP condition (Figure 4biii), there were only 45 upregulated genes involved in transcription regulator activity, specifically transcription factor binding (e.g., *HMGB2*, *SALL4*, *RORA*, *HNF4A*) that involved STAT family protein binding (e.g., *CEBPA*, *SPI1*), central nervous system differentiation (e.g., *NKX2-2*, *NKX6-1*, *TAL2*, *GLI1*, *HOXA1*) (data not shown). Downregulated genes in 100 μM NIMP relative to 4 μM NIMP condition included processes such as negative regulation of cell communication, positive regulation of cell differentiation, negative regulation of the immune system process, positive regulation of monoatomic ion transport, and negative regulation of hormone secretion, cell adhesion and the extracellular matrix.

**FIGURE 5:**
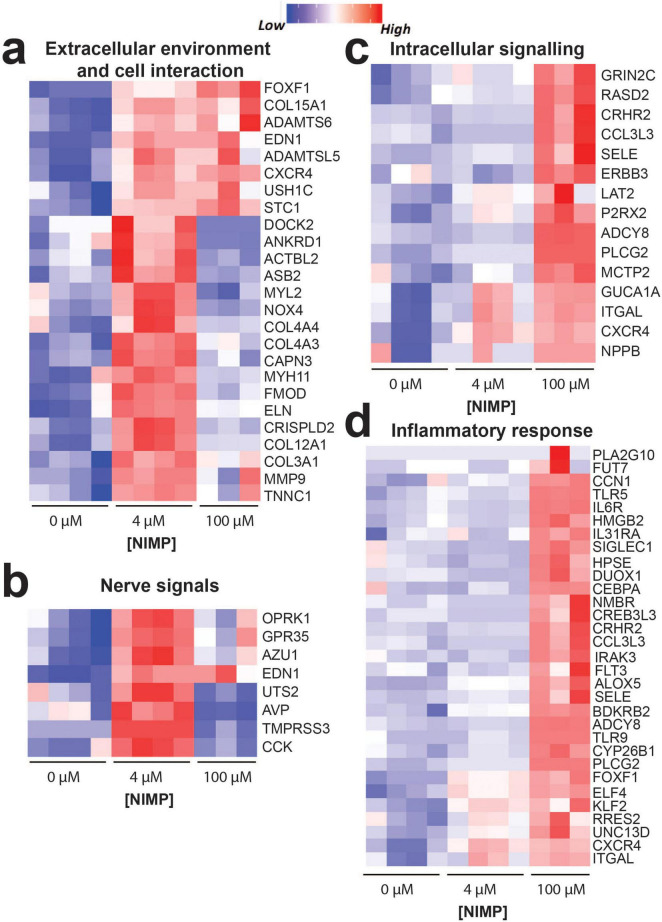
Key differentially expressed genes. Heatmap of genes showing the upregulation or downregulation of genes from human iPSC-derived neurons co-cultured with primary human astrocytes exposed at 6 days post NIMP exposure. Biological processes include extracellular environment and cell interaction **(**a**)**, nerve signals **(**b**)**, intracellular signaling **(**c**)**, and inflammatory response **(**d**)**.

In Figure 5, we highlighted genes that were involved in processes for the extracellular environment and cell interaction, nerve signals, intracellular signaling, and the inflammatory response. For extracellular environment and cell interaction (Figure 5a), genes relevant to cell morphogenesis (*EDN1*, *USH1C*, *COL15A1*, *CXCR4*, and *STC1*) were upregulated in both the 4 and 100 μM NIMP conditions. Several genes involved in collagen fibril organization (e.g., *COL3A1*, *COL12A1*, *FMOD*, and *MMP9*), cytoskeletal organization (e.g., *DOCK2*, *NOX4*, *NYH11, ACTBL2*, *ELN*, *ANKRD1*, *MYL2*, *CAPN3*, and *ASB2*), and nerve signals were upregulated only in the 4 μM NIMP but not 100 μM treatment condition relative to 0 μM. In particular to nerve signals (Figure 5b), upregulated genes in the 4 μM NIMP condition were involved in protein kinase C-activating (e.g., *EDN1*, *CCK*, *AZU1*), phospholipase C-activating G protein-coupled receptor (e.g., *EDN1*, *OPRK1*, *GPR35*) signaling pathways and sodium homeostasis (e.g., EDN1, AVP, *TMPRSS3*, *UTS2*). For intracellular signaling (Figure 5c), upregulated genes in only the 100 μM NIMP condition were involved in cGMP-mediated signaling (e.g., GUCA1A and NPPB), cAMP-mediated signaling (e.g., *ADCY8*, *CRHR2*, *RASD2*) and calcium-mediated signaling (e.g., *GRIN2C*, *SELE*, *ITGAL*, *MCTP2*, *P2RX2*, *CXCR4*, *ERBB3*, *LAT2*, *CCL3L3*, *PLCG2*). In addition, genes involved in the modulation of chemical synaptic transmission (e.g., *MCTP2*, *P2RX2*, *ADCY8*, *CRHR2*, and *GRIN2C*) were also upregulated in only the 100 μM NIMP condition. For inflammation (Figure 5d), there was a subset of genes that did not meet the fold change and *p*-value cutoff but was trending toward upregulation in the 4 μM NIMP condition and was significant in the 100 μM NIMP condition. These genes were involved in the regulation of the immune system process (e.g., *ITGAL*, *FOXF1*, *CXCR4*, *UNC13D*, and *RARRES2*). In addition, genes involved in IL-6, type I interferon, and/or tumor necrosis factor production (e.g., *IRAK3*, *ELF4*, *FLT3*, *PLCG2*, *TLR9*, *NMBR*, *CCN1*, *IL6R*, *CRHR2*, *KLF2*, and *TLR5*) were upregulated in the 100 μM NIMP condition.

We also observed significant expression changes (*p* < 0.05) for genes encoding neurotransmitter receptor subunits that did not meet the FC > 1.5 criteria but exhibited at least 1.25 fold difference (i.e., log2FC > 0.322) and may be sufficient to modulate neural and network activity. These included receptor subunits for GABA_*A*_ receptors (e.g., *GABRR1*, *GABRE*, *GABRD*) that are upregulated in the low (but not high) concentration of NIMP, and glutamate ionotropic receptor NMDA type subunit (e.g., *GRIN2C*) and muscarinic acetylcholine receptor subunit (e.g., *CHRM5*) that is upregulated in both low and high concentrations of NIMP.

## 4 Discussion

In the present study, there were several salient findings when we exposed the human-relevant MEA system to the sarin-surrogate NIMP and monitored the functional consequences on human iPSC-derived neurons and their established networks. (i) The concentration-response study revealed a biphasic response in specific features of spiking (but not bursting) activity described by the hyperexcitability during exposure to low concentrations of NIMP (0.4 and 4 μM) not observed during exposure to high concentrations of NIMP (40 and 100 μM). Further, we report a decline in neural excitability at 24 h of NIMP exposure and up to 2 days post-exposure for cultures exposed to the high but not low concentrations of NIMP. (ii) Synchrony analysis revealed that only a subset of neural networks was affected for both low and high concentrations of NIMP during the period of exposure. However, only networks previously exposed to a high concentration of NIMP had shown a change in the degree of synchronization at 6 days post-exposure. (iii) Catalytic activity from soluble and membrane-bound AChE verified NIMP-induced inhibition of AChE activity during the 24 h period of exposure, and the enzyme activity recovered to untreated levels by 6 days post NIMP exposure. Lastly, (iv) transcriptomic data revealed gene expression changes relevant to the cell’s interaction with the extracellular environment, its intracellular calcium signaling pathways (e.g., PLC and PKC) and inflammation. Collectively, our study has found that a single exposure to NIMP can lead to short-term alterations (e.g., days following exposure) in network activity and the transcriptome of human iPSC-derived neurons and primary human astrocytes. However, the concentration of exposure suggests differences in the mechanism of action (e.g., AChE dependence, changes in intracellular calcium signaling pathways, and inflammation) that will need to be explored in future studies.

A concentration-dependent effect of organophosphates on neural and network activity *in vitro* has been reported for organophosphate (OP) insecticides (e.g., chlorpyrifos, diazinon, and their respective -oxon metabolites) (van Melis et al., 2023; van Melis et al., 2024). Acute and chronic exposure to insecticides at concentrations ranging from 0.1 to 100 μM was shown to decrease neural and network activity when concentrations were greater than 10 μM of the OP insecticides. To our knowledge, reports of a concentration-dependent effect of NIMP on neural activity remain scarce. In one study, Thinschmidt et al. (2022) reported a concentration-dependent effect at low micromolar concentrations of NIMP on rat basolateral amygdala pyramidal neurons isolated from acute rat brain slices using whole-cell patch clamp technique (Thinschmidt et al., 2022). In particular, enhanced response in the electrical properties of the neuron [e.g., the holding current, spontaneous excitatory postsynaptic current (sEPSC) frequency, and amplitude] was observed following ≥ 2 h preincubation with 0.3 and 10 μM NIMP that was not observed from slices preincubated with 0.1 μM NIMP. Further, the authors demonstrated this enhanced response or hyperexcitability was mediated by the M1 muscarinic acetylcholine receptor. In the current study, we used concentrations that were comparable to Thinschmidt et al. (2022), specifically 0.4 and 4 μM, and also report hyperexcitability of human-iPSC derived neurons that is described by increase in specific features of spiking (e.g., firing rate and total number of spikes, Figure 1 and Supplementary Figure 2) but not bursting activity. At higher concentrations of NIMP (e.g., 40 and 100 μM), we did not observe an increase in neuronal excitability during 24 h of NIMP exposure, but rather a decline in spiking activity (e.g., firing rate and total number of spikes) as early as 24 h of NIMP exposure that persisted up to 2 days post-exposure. Notably, by 6 days post-exposure, altered features of spiking activity returned to levels comparable to 0 μM NIMP for both “low” and “high” concentrations of NIMP. Resolution of neural activity was also observed from neurons isolated from the hippocampal tissues of guinea pigs exposed to a single dose of soman, in which the increase in frequency of sEPSC observed after 24 h of NIMP exposure had resolved by 7 days (Harrison et al., 2005). However, we report that the communication or coordination of activity between neurons at the network level {combining synchrony analysis (Figure 2a) and statistical methods [e.g., QQ plots (Figure 2b) and KL divergence (Figure 2c)]} did not resolve for the “high” concentrations of NIMP by 6 days post-exposure as it did for cultures exposed to the “low” concentrations of NIMP. This suggested that a single 24 h exposure to NIMP induced lasting changes to the coordination of neural activity from human iPSC-derived neurons.

The primary mode of action for organophosphates is AChE inhibition. For NIMP, the half maximal inhibitory concentration (IC_50_) for human erythrocyte AChE was 43 nM in a cell-free assay (Meek et al., 2012). The NIMP concentrations used in the present study (e.g., 0.4–100 μM) were greater than the reported IC_50_ for human AChE. We have shown that these concentrations inhibit both the soluble (∼40–50%) and membrane bound AChE (26 and 35%) collected from the culture supernatant and cell lysate of the human-relevant MEA system during and/or after NIMP exposure (Figure 3aii). In rodent studies, AChE IC_50_ for brain tissue has been shown to be a good predictor of the median lethal dose (LD_50_) values for nerve agents in experimental animal studies (Meek et al., 2012; Fawcett et al., 2009; Khan et al., 2000). Thus, the concentrations used in this study are potentially sub-lethal potencies that differentially affect human-relevant neural networks. Interestingly, we found that the degree of AChE inhibition was not concentration-dependent (Figure 3a) despite the concentration-dependent response observed from neural and network activity. Previous concentration-response studies for neuronal cultures exposed to NIMP or OP insecticides had not evaluated the relationship between the degree of AChE inhibition and the changes in neural activity. Within the range of our low micromolar concentrations (0.4 and 4 μM) of NIMP, Thinschmidt et al. (2022) reported that hyperexcitability induced at 0.3 and 10 μM NIMP was dependent on acetylcholine binding to M1 muscarinic acetylcholine receptors expressed in rat basolateral amygdala pyramidal neurons isolated from acute rat brain slices. However, they did not determine the degree of AChE inhibition in this preparation. Within range of our high micromolar concentration (40 and 100 μM) of NIMP, two studies reported that primary rat cortical and human SH-SY5Y exposed to high concentrations of OP insecticides showed reduced neuronal activity and inhibition of depolarization-evoked calcium influx that was not attributed to cell viability (van Melis et al., 2023; van Melis et al., 2024). Measurement of AChE activity was also not conducted in this study. Our study showed a similar decline in neural activity that occurred days following exposure to a high concentration of NIMP and agreed with the OP insecticide studies that cell viability was unaffected (Figure 3b). We demonstrated that measurement of AChE inhibition from the human-relevant MEA system does report restored AChE activity to levels comparable to wells from the untreated condition. OP insecticide exposure studies have recently postulated OP-mediated non-AChE mechanisms (van Melis et al., 2023; van Melis et al., 2024) where NIMP can directly modulate voltage-gated calcium channels (Meijer et al., 2015; Meijer et al., 2014). Another group has computationally predicted non-AChE targets (Souza et al., 2023) since experimental data is limited. While non-AChE mechanism remains poorly understood, the collective findings from our study and in previous concentration-response studies indicate that there is still the need for pharmacological studies to investigate the direct mode(s) of action of NIMP (e.g., AChE and non-AChE mechanisms) within the concentration-dependent response in neural activity.

Gene expression analysis (Figures 4, 5) of human iPSC-derived neurons and primary human astrocytes from our system revealed that a single exposure to NIMP (at 4 and 100 μM) also revealed a concentration-dependent response in the transcriptome of cells. Specifically, differential genes expressed in the 4 vs. 100 μM NIMP condition suggest a difference in signaling pathways used to regulate intracellular calcium levels (e.g., PLC, PKC, second messenger molecules) (Callender and Newton, 2017; Halls and Cooper, 2011; Putney and Tomita, 2012). For the 4 μM NIMP condition, upregulated genes were involved in protein kinase C-activating and phospholipase C (PLC)-activating G protein-coupled receptor signaling pathways that was not altered in 100 μM NIMP condition. Previous studies from seizing rats exposed to sarin, soman, or their surrogates have reported a time-dependent (from 30 min to 2 h) increase in the phosphorylation of the isozymes PLCβ and γ and enzyme activity (Niijima et al., 1999), and the upregulation of genes relevant to PLC signaling in the thalamus at 24 h of exposure (Spradling et al., 2011). Similarly, brain region-specific regulation of PKC isozymes, βII-PKC ζ-PKC, were found hours to days following sarin and soman exposure (Bloch-Shilderman et al., 2005; RamaRao et al., 2011). It is interesting that genes relevant to these intracellular signaling pathways are still upregulated 6 days after exposure to a low concentration of NIMP found to induce neuronal hyperexcitability during the 24 h period of exposure. In contrast, the 100 μM NIMP condition upregulated genes that involved second messenger molecules, calcium, cGMP- and cAMP that were not differentially regulated in the 4 μM NIMP condition. A limited number of studies report the role of cyclic nucleotides following OPNA or NIMP exposure. In two studies, cGMP was found elevated in the rat cerebellum at the onset and duration of convulsions during soman (at concentrations 1 or 2x the LD_50_) exposure, which were lowered or blocked when rats were treated with certain anticonvulsants (Lundy and Shaw, 1983; Lundy and Magor, 1978). In another study, the upregulation of genes related to cAMP signaling was detected in the brain tissue of rats who had recovered for 21 following chronic exposure to low level sarin (0.4x the LD_50_) for 21 days (Shi et al., 2023). In the present study, it is unclear how intracellular calcium levels compare to the changes in spiking activity observed in the low and high concentrations of NIMP during the period of exposure and post-exposure. Various OPs have been shown to increase intracellular calcium levels (see review Costas-Ferreira and Faro, 2021), and it is notable that in the present study certain signaling molecules known to regulate intracellular calcium are dependent on the concentration of NIMP. It will be important for future studies to examine whether the recruitment of different signaling pathways (e.g., Ca^2+^-, PKC-, PLC, cAMP-, and cGMP-dependent) to increasing concentrations of NIMP is needed to modulate intracellular calcium levels to maintain homeostatic spiking activity post-exposure as observed in the present study. Other genes found that could contribute to the change in neural and network activity observed also included genes involved in the modulation of chemical synaptic transmission and for specific neurotransmitter receptor subunits, which were differentially regulated at > 1.5 or 1.25 fold change, respectively, from human-relevant MEA system exposed to 0 μM NIMP. Thus, additional studies will be needed to determine whether the reported changes in neurotransmitter receptor subunit expression affect the kinetics and/or pharmacology of GABA_A_ receptors, glutamate ionotropic NMDA receptors, and muscarinic acetylcholine receptors that modulate neuronal and network activity during and following NIMP exposure.

Lastly, gene expression analysis also detected a greater inflammatory response in the human-relevant MEA system exposed to a high concentration (e.g., 100 μM) of NIMP, void of immune cells (e.g., innate or peripheral immune cells). These genes were involved in regulation of the immune response process and production of pro-inflammatory cytokines (e.g., IL-6, type I interferon, and tumor necrosis factor). Both transcripts and protein levels of IL-6 and TNF-α have been detected and elevated in multiple brain regions of rats and mice acutely exposed to soman and sarin and resolved by 1–2 days post-exposure [see review (Banks and Lein, 2012)]. A second wave of pro-inflammatory cytokine (e.g., IL-6, TNF-α, and IL-1β) was detected 1 month later in the cortex of rats that had a prolonged seizure following acute exposure to sarin (Chapman et al., 2006), which also suggests lasting effects following an acute exposure. The primary human astrocytes within our system are a potential source of these pro-inflammatory cytokines and are potentially recruited to modulate neural activity when exposed to higher concentrations of NIMP. In one study, primary human astrocytes treated for 1 week with OP insecticides, cyfluthrin or chlorpyrifos, were shown to upregulate genes and protein levels for IL-6 and genes involved in the signaling pathway for type II interferon, IFN-γ (Mense et al., 2006). Thus, astrocytes in the human-relevant MEA system can also contribute to regulating neural and network activity, and inducing, or modulating the inflammatory response.

Collectively, this concentration-response study provided a non-invasive and real-time analysis of the functional dynamics of human iPSC-derived neural and network activity during the 24 h of NIMP exposure and up to 6 days post-exposure. Our findings revealed a concentration-dependent biphasic response in spiking activity that had disrupted the synchronization of neurons at a network level. Further analysis of the catalytic activity of AChE and transcriptomic data suggests potential mechanisms that may regulate the biphasic response during and shortly after NIMP exposure. More specifically, these are genes involved in signaling pathways that regulate intracellular calcium levels and inflammation. Thus, it will be important for future studies to evaluate the direct (e.g., AChE and non-AChE) and downstream mechanisms that contribute to the biphasic response of NIMP on neural and network activity that will enable a more targeted therapeutic strategy or identify therapeutic windows that will protect neuronal function and prevent the disruption of the coordinated neural network activity observed.

## Data Availability

The datasets presented in this study can be found in online repositories. The names of the repository/repositories and accession number(s) can be found below: https://www.ncbi.nlm.nih.gov/geo/, GSE251844.

## Footnotes

1
https://www.bioinformatics.babraham.ac.uk/projects/fastqc/

2
https://usegalaxy.eu/

## Supplementary material

Supplementary Figure 1 Percentage of active electrodes detected on the human-relevant MEA system. The human-relevant MEA system containing human iPSC-derived glutamatergic and GABAergic neurons co-cultured with primary human astrocytes showed active electrodes that detected action potential spikes at 14 DIV. The percentage of active electrodes stabilized over the period of 25 DIV before NIMP exposure. Data is shown as mean ± SEM for *n* = 84 human-relevant MEA systems or 14 devices in the 6-well format. Statistical analysis was conducted using mixed model repeated measures two-way ANOVA with Tukey’s *post-hoc* test to compare time points. Statistical significance is shown at a level of ***p* < 0.01 and *****p* < 0.0001.

Supplementary Figure 2 Other features of spiking and bursting activity from human iPSC-derived neurons co-cultured with primary human astrocytes during 24 h of NIMP exposure and up to 6 days post-exposure. Bar graph summarizes the percentage of active electrodes, and other features of spiking (e.g., total number of spikes) and bursting (e.g., total number of bursts and interburst interval [IBI]) before (e.g., baseline [b]), during (e.g., 30 and 60 min, 24 h), and after (e.g., immediately after washout [24 h-W], 1-[D1], 2-[D2], and 6-[D6] days) NIMP (e.g., 0, 0.4, 4, 40, and 100 μM) exposure. Data is shown as the difference in the mean value for the treatment condition relative to the age-matched 0 μM NIMP condition, shown as mean ± SEM for *n* = 7–13 wells/ treatment condition. Statistical analysis was conducted using mixed model repeated measure two-way ANOVA with Tukey’s *post-hoc* test to compare age-matched treated and 0 μM NIMP conditions (*). Statistical significance is shown at a level of **p* < 0.05.

Supplementary Figure 3 Distribution of synchrony scores from human iPSC-derived neural networks before, during, and after NIMP exposure. Histograms report the probability of synchrony scores from a pair of electrodes (or network) detected across all human-relevant MEA systems within a specific time point before (e.g., baseline), during (e.g., 30 min, 60 min, and 24 h, **ai**), and after (e.g., 24 h immediate washout, 1-, 2-, and 6 days post-exposure, **aii**). Data is shown for each NIMP concentration and corresponding sample size: 0 μM (black, *n* = 12 human-relevant MEA system), 0.4 μM (green, *n* = 7), 4 μM (yellow, *n* = 8), 40 μM (light blue, *n* = 11) and 100 μM (dark blue, *n* = 13). In panels, **(bi,bii)**, we show the empirical cumulative probability distribution of the data from **(ai,aii)**. For readability in **(bi,bii)** we focus the plot on the synchrony range between 0.2 and 0.8.
