# Supplementary figures and images for: Biphasic response of human iPSC-derived neural network activity following exposure to a sarin-surrogate nerve agent
Source: Front Cell Neurosci. 2024 Sep 5;18:1378579. doi: 10.3389/fncel.2024.1378579 (PMC11410629; doi:10.3389/fncel.2024.1378579)

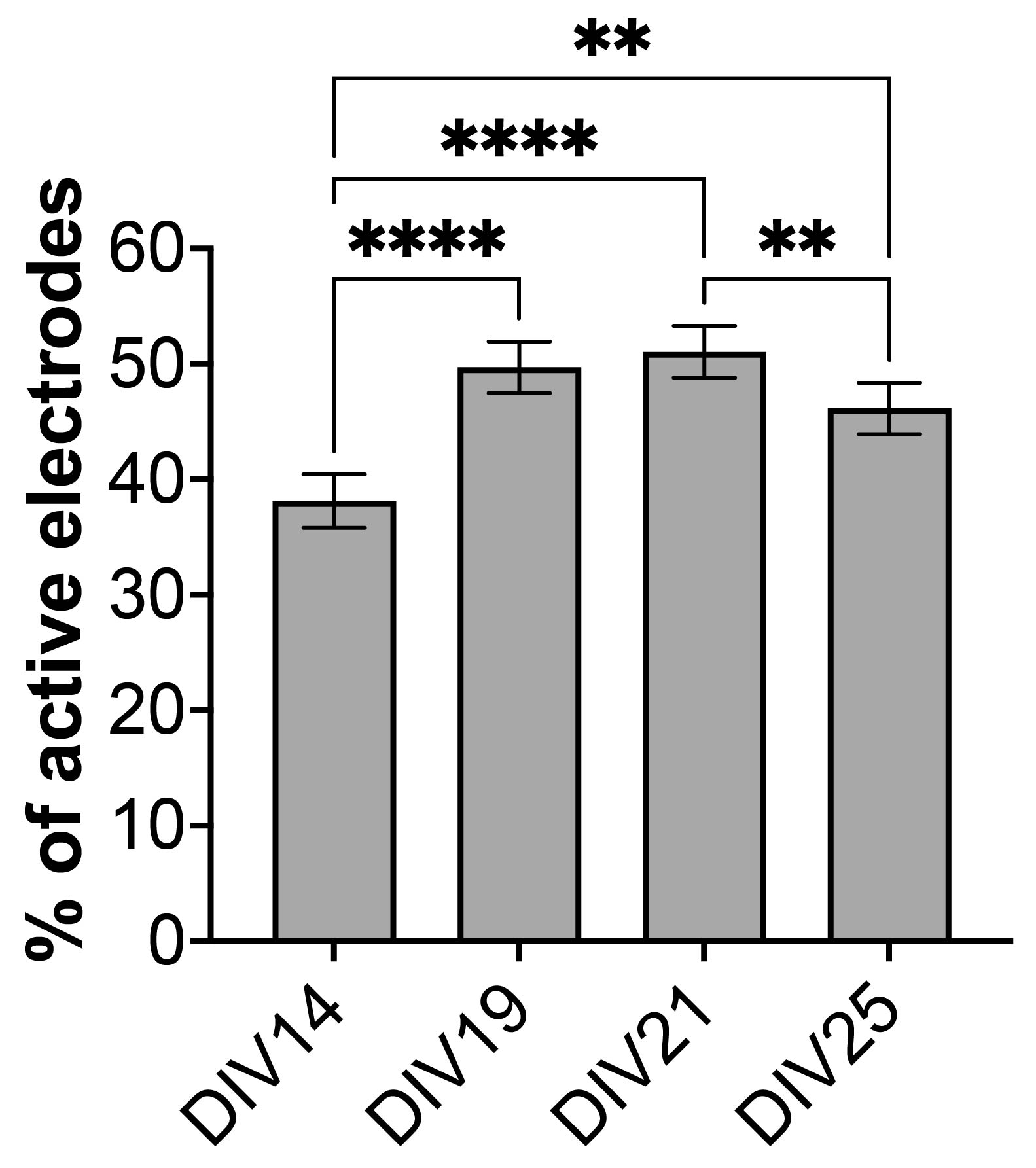

Supplement: Supplementary Figure 1 — Percentage of active electrodes detected on the human-relevant MEA system. The human-relevant MEA system containing human iPSC-derived glutamatergic and GABAergic neurons co-cultured with primary human astrocytes showed active electrodes that detected action potential spikes at 14 DIV. The percentage of active electrodes stabilized over the period of 25 DIV before NIMP exposure. Data is shown as mean ± SEM for n = 84 human-relevant MEA systems or 14 devices in the 6-well format. Statistical analysis was conducted using mixed model repeated measures two-way ANOVA with Tukey’s post-hoc test to compare time points. Statistical significance is shown at a level of **p < 0.01 and ****p < 0.0001. [file Image_1.JPEG]

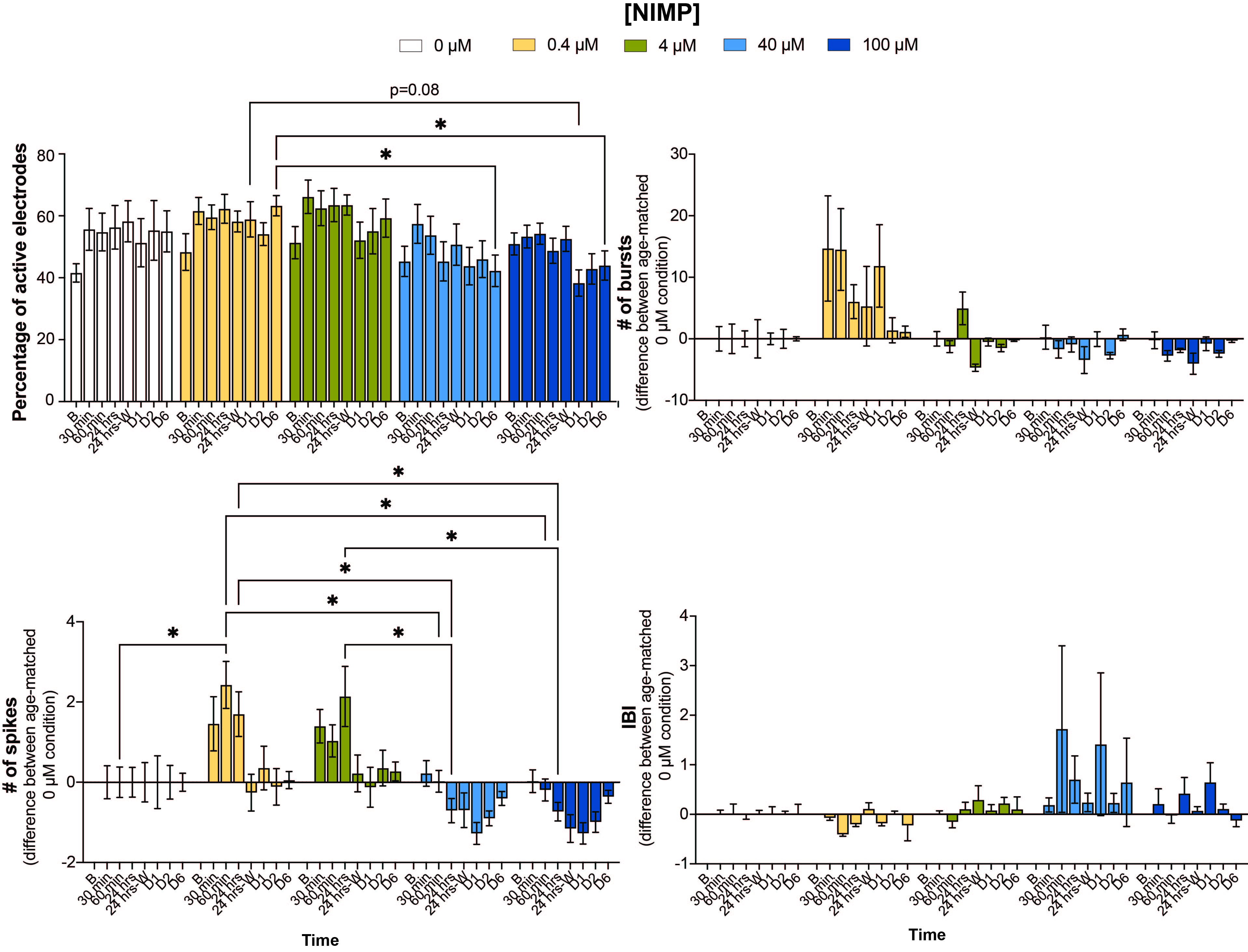

Supplement: Supplementary Figure 2 — Other features of spiking and bursting activity from human iPSC-derived neurons co-cultured with primary human astrocytes during 24 h of NIMP exposure and up to 6 days post-exposure. Bar graph summarizes the percentage of active electrodes, and other features of spiking (e.g., total number of spikes) and bursting (e.g., total number of bursts and interburst interval [IBI]) before (e.g., baseline [b]), during (e.g., 30 and 60 min, 24 h), and after (e.g., immediately after washout [24 h-W], 1-[D1], 2-[D2], and 6-[D6] days) NIMP (e.g., 0, 0.4, 4, 40, and 100 μM) exposure. Data is shown as the difference in the mean value for the treatment condition relative to the age-matched 0 μM NIMP condition, shown as mean ± SEM for n = 7–13 wells/ treatment condition. Statistical analysis was conducted using mixed model repeated measure two-way ANOVA with Tukey’s post-hoc test to compare age-matched treated and 0 μM NIMP conditions (*). Statistical significance is shown at a level of *p < 0.05. [file Image_2.JPEG]

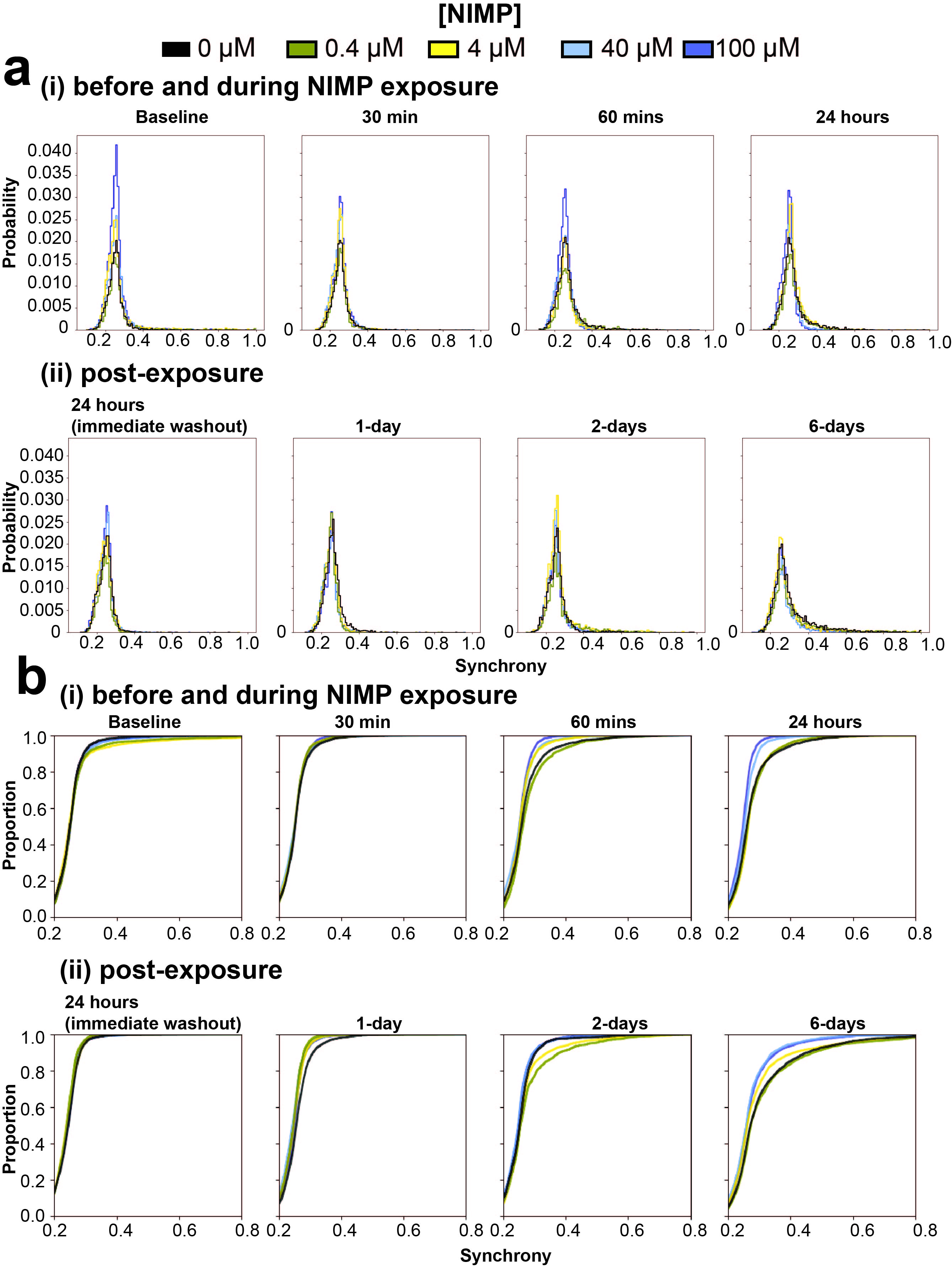

Supplement: Supplementary Figure 3 — Distribution of synchrony scores from human iPSC-derived neural networks before, during, and after NIMP exposure. Histograms report the probability of synchrony scores from a pair of electrodes (or network) detected across all human-relevant MEA systems within a specific time point before (e.g., baseline), during (e.g., 30 min, 60 min, and 24 h, ai), and after (e.g., 24 h immediate washout, 1-, 2-, and 6 days post-exposure, aii). Data is shown for each NIMP concentration and corresponding sample size: 0 μM (black, n = 12 human-relevant MEA system), 0.4 μM (green, n = 7), 4 μM (yellow, n = 8), 40 μM (light blue, n = 11) and 100 μM (dark blue, n = 13). In panels, (bi,bii), we show the empirical cumulative probability distribution of the data from (ai,aii). For readability in (bi,bii) we focus the plot on the synchrony range between 0.2 and 0.8. [file Image_3.JPEG]
